# Development and Application of SSR Markers Related to Genes Involved in Leaf Adaxial-Abaxial Polarity Establishment in Chinese Cabbage (*Brassica rapa* L. ssp. *pekinensis*)

**DOI:** 10.3389/fgene.2020.00773

**Published:** 2020-07-23

**Authors:** Ying Gao, Yin Lu, Xiaoguang Li, Na Li, Xiaomeng Zhang, Xiangjie Su, Daling Feng, Mengyang Liu, Shuxin Xuan, Aixia Gu, Yanhua Wang, Xueping Chen, Jianjun Zhao, Shuxing Shen

**Affiliations:** ^1^State Key Laboratory of North China Crop Improvement and Regulation, Key Laboratory of Vegetable Germplasm Innovation and Utilization of Hebei, Collaborative Innovation Center of Vegetable Industry in Hebei, College of Horticulture, Hebei Agricultural University, Baoding, China; ^2^Agriculture and Rural Affairs Bureau of Xindu District, Xingtai, China; ^3^Institute of Forestry and Fruits, Xingtai Academy of Agricultural Sciences, Xingtai, China

**Keywords:** Chinese cabbage, leaf ad-ab polarity, SSR, genetic linkage map, marker-assisted selection

## Abstract

In Chinese cabbage (*Brassica rapa* L. ssp. *pekinensis*), leaf adaxial-abaxial (ad-ab) polarity is tightly related to leaf incurvature, an essential factor for the formation of leafy heads. Therefore, identification of the genes responsible for leaf ad-ab polarity and studying their genetic variation may clarify the mechanism of leafy head formation. By comparing the sequences of the genes regulating leaf ad-ab polarity development in *Arabidopsis thaliana* (*A. thaliana*), 41 candidate genes distributed on 10 chromosomes were found to be responsible for the establishment of ad-ab polarity in Chinese cabbage. Orthologous genes, including 10 single copies, 14 double copies, and one triple copies, were detected in the Chinese cabbage. The gene structure and conserved domain analyses showed that the number of exons of the 41 candidate genes range from one to 25, and that most genes share the conserved motifs 1, 6, and 10. Based on the 41 candidate genes, 341 simple sequence repeats (SSRs) were detected, including five replicated types: single, double, triple, quintuple, and sextuple nucleotide replications. Among these sequence repeat (SSR) loci, 323 loci were used to design 969 specific primers, and 362 primer pairs were selected randomly and evaluated using 12 Chinese cabbage accessions with different heading types. 23 primer pairs resulting with clear, polymorphic bands, combined with other 127 markers, was used to construct a linkage map by using an F_2_ population containing 214 lines derived from the hybrid of the overlapping heading Chinese cabbage “14Q-141” and the outward curling heading Chinese cabbage “14Q-279.” The result showed that the sequences of markers in the genetic linkage map and the physical map was consistent in general. Our study could help to accelerate the breeding process of leafy head quality in Chinese cabbage.

## Introduction

Chinese cabbage (*Brassica rapa* L. ssp. *pekinensis*) is an important leafy vegetable grown worldwide and one of the most consumed vegetables in Asia. With the improvement of life quality, the leafy head appearance of Chinese cabbage is of increasing concern to both the consumers and breeders (Mao et al., 2014; Liang et al., 2016). Chinese cabbage goes through three developmental stages to produce a leafy head, namely, seedling, rosette, and heading (Yu et al., 2013). The leaves grow flat in the seedling and rosette stages, whereas in the heading stage they curve inwardly and show a large abaxial surface. Leaf incurvature is influenced by leaf ad-ab polarity, and is a precondition for the formation of leafy heads (Mao et al., 2014; Liang et al., 2016). Therefore, studies on leaf ad-ab polarity is helpful for improving the commercial traits of Chinese cabbage (Yu et al., 2013; Mao et al., 2014).

Leaf heading is a complicated quantitative trait controlled by various of genes (Yu et al., 2013; Wang et al., 2014). With the completion of the whole genome sequencing of the model plant *A. thaliana* and a variety of other plants (Initiative, 2000; Wang et al., 2011), as well as the deep research in molecular biology and genetics, it has been found that the regulatory network controlling ad-ab polarity mainly involves abaxial patterning genes, adaxial patterning genes, *WUSCHEL-RELATED HOMEOBOX* (*WOX*) genes, *YABBY* genes, and small RNAs genes (Yamaguchi et al., 2012). In angiosperm models, the adaxial polarity is regulated by genes of the class III homeodomain-leucine zipper (HD-ZIPIII) family [*REVOLUTA* (*REV*), *ATHB8*, *PHAVOLUTA* (*PHV*), and *PHB*] (Mcconnell et al., 2001; Emery et al., 2003), Myb, and LOB domain transcription factors *ASYMMETRIC LEAVES1* (*AS1*) and *AS2* (Lin et al., 2003; Iwakawa et al., 2007). In contrast, the abaxial polarity is specified by the *AUXIN RESPONSE FACTORS* (*ARF3* and *ARF4*) (Pekker et al., 2005), *KANADI* family genes (*KAN1*, *KAN2*, and *KAN3*) (Eshed et al., 2001, 2004; Kerstetter et al., 2001), and small RNAs miR165/166 (Kidner and Martienssen, 2004). *WUS-related homeobox* (*WOX*) genes (*WOX1*, *WOX3*), and *YABBY* genes are involved in adaxial/abaxial patterning and subsequent flat leaf growth (Kidner and Timmermans, 2007; Vandenbussche et al., 2009; Nakata et al., 2012). In addition, leaf ad-ab polarity is also regulated by microRNAs and tasiRNAs, which include *ARGONAUTE1* (*AGO1*), *ARGONAUTE7* (*AGO7*), RNA-*DEPENDENT RNA POLYMERASE 6* (*RDR6*), *SUPPRESSOR OF GENE SILENCING 3* (*SGS3*), *SERRATE* (*SE*), *DICER-LIKE1* (*DCL1*) and *DICER-LIKE4* (*DCL4*) (Moon and Hake, 2011). Furthermore, 45 genes (Version 1.5) involved in leaf adaxial-abaxial polarity establishment were detected in Chinese Cabbage (Liang et al., 2016). Chinese cabbage and *Arabidopsis thaliana* both belong to Cruciferae, giving the collinearity of the two genomes, most genes involved in leaf ad-ab polarity establishment are concluded to be conserved between these two genome.

Developing and utilizing molecular markers related to leaf ad-ab polarity establishment may lead to a better marker-assisted selection in breeding. SSR or microsatellites, are iterations of 1–6 bp nucleotide motifs. SSR molecular markers are widely distributed in genomes, have a high level of polymorphisms, are inherited co-dominantly and can be easily analyzed by PCR. Therefore, it becomes one of the most popular molecular markers and have been used widely to construct genetic linkage map, indentify varieties, and carry out diverse genetic analyses (Zhao et al., 2008; Gao et al., 2012). With the accomplishment of genome sequencing in Chinese cabbage (Wang et al., 2011), it is possible to develop the molecular markers related to leaf ad-ab polarity based on the information of regulators from *Arabidopsis*.

In this study, we developed specific SSR markers that correlate with the establishment of leaf ad-ab polarity. The repeat units and distribution characteristics of these SSR loci as well as the amplification efficiency and polymorphism level were analyzed. Our study will benefit to clarify the molecular mechanism of leafy head formation, and help to accelerate breeding process in leafy head quality in Chinese cabbage.

## Materials and Methods

### Plant Materials

**FIGURE 1:**
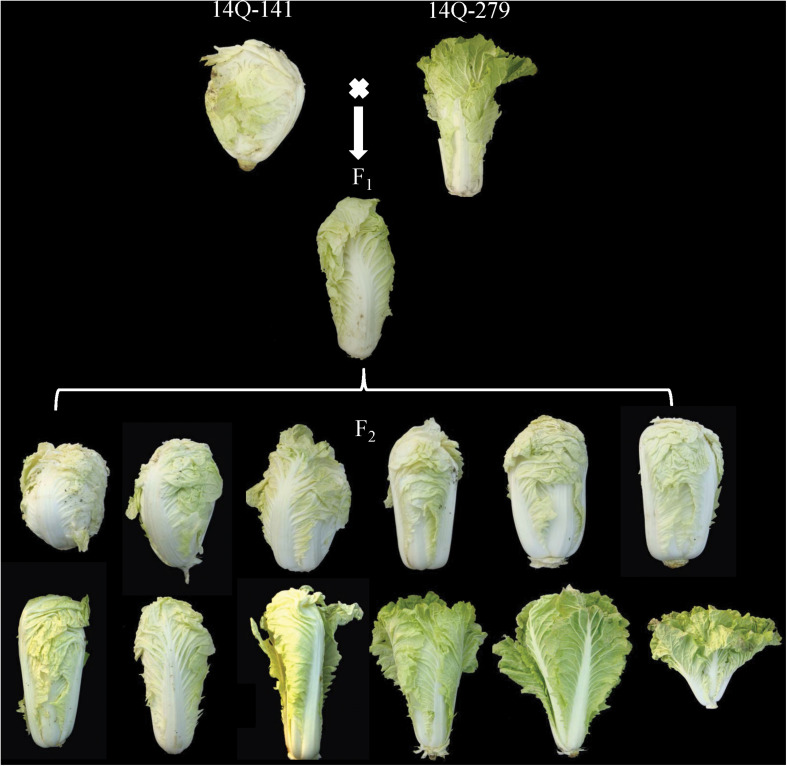
Parents, F_1_ and representatives in F_2_ population.

An F_2_ population was used as the mapping population in this study. The female parent of the population was the overlapping heading Chinese cabbage “14Q-141,” and the male parent was the outward curling heading Chinese cabbage “14Q-279” (Figure 1). F_1_ was obtained from a cross between “14Q-141” and “14Q-279.” 214 F_2_ plants were gained after F_1_ selfing. The experiment was carried out at Hebei Agricultural University in Hebei, China. The seeds of the 214 F_2_ and their parental lines were sown in greenhouse and the seedlings were transplanted to an open field in September 2016, and kept growing until November 2016 in natural conditions.

### The Sequences Updates of Genes Responsible for Leaf ad-ab Polarity in Chinese Cabbage

The sequences of genes responsible for leaf ad-ab polarity in Chinese cabbage was published in version 1.5 (Liang et al., 2016). The BRAD database^1^ (Cheng et al., 2011) was used to update the sequences information of these genes from version 1.5 to version 3.0. Table 1 showed the genes responsible for the establishment of leaf ad-ab polarity in Chinese cabbage.

**TABLE 1:** Leaf ad-ab polarity–controlling genes in Chinese cabbage.

Regulation pathway	Gene	*Arabidopsis thaliana* genes	Chinese cabbage genes	Location in Linkage group
Adaxial determinants	*REV*	AT5G60690	*BrREV1* (BraA10g018460.3C)	chr10: 13671299..13675382
			*BrREV2* (BraA02g010200.3C)	chr02: 4908251..4904176
	*PHB*	AT2G34710	*BrPHB1* (BraA05g010360.3C)	chr05: 5534983..5539344
			*BrPHB2* (BraA04g024760.3C)	chr04: 17922205..17918117
	*PHV*	AT1G30490	*BrPHV* (BraA09g034560.3C)	chr09: 27178071..27182489
	*ATHB8*	AT4G32880	*BrHB8.1* (BraA01g005160.3C)	chr01: 2462816..2467248
			*BrHB8.2* (BraA08g016820.3C)	chr08: 13619843..13624628
	*AS1*	AT2G37630	*BrAS1.1* (BraA05g007920.3C)	chr05: 4047371..4048456
			*BrAS1.2* (BraA03g019630.3C)	chr03: 9309263..9308220
	*AS2*	AT1G65620	*BrAS2* (BraA02g016770.3C)	chr02: 8953550..8954158
Middle domain determinants	*YAB1*	AT2G45190	*BrYAB1.1* (BraA04g030760.3C)	chr04: 21056868..21059180
			*BrYAB1.2* (BraA03g023630.3C)	chr03: 11342365..11340257
	*YAB2*	AT1G08465	*BrYAB2.1* (BraA06g005620.3C)	chr06: 3309705..3311007
			*BrYAB2.2* (BraA08g033150.3C)	chr08: 21874379..21873237
			*BrYAB2.3* (BraA09g062760.3C)	chr09: 43480055..43477632
	*YAB3*	AT4G00180	–	–
	*YAB5*	AT2G26580	*BrYAB5* (BraA03g025390.3C)	chr03: 12518810..12521194
	*WOX1*	AT3G18010	*BrWOX1.1* (BraA05g029540.3C)	chr05: 21857860..21859470
			*BrWOX1.2* (BraA03g038230.3C)	chr03: 18917799..18915771
	*WOX3*	AT2G28610	*BrWOX3.1* (BraA04g020450.3C)	chr04: 15389303..15390390
			*BrWOX3.2* (BraA03g024810.3C)	chr03: 12196864..12195825
Abaxial determinants	*KAN1*	AT2G28610	*BrKAN1* (BraA10g023200.3C)	chr10: 16161361..16168134
	*KAN2*	AT1G32240	*BrKAN2.1* (BraA09g032840.3C)	chr09: 25471282..25477168
			*BrKAN2.2* (BraA05g023490.3C)	chr05: 17380156..17384804
	*KAN3*	AT4G17695	*BrKAN3.1* (BraA01g009320.3C)	chr01: 4661501..4658601
			*BrKAN3.2* (BraA08g012540.3C)	chr08: 10833475..10830673
	*ARF3*	AT2G33860	*BrARF3.1* (BraA05g011080.3C)	chr05: 5950051..5952569
			*BrARF3.2* (BraA04g024390.3C)	chr04: 17725613..17723081
	*ARF4*	AT5G60450	*BrARF4.1* (BraA10g018230.3C)	chr10: 13539330..13535905
			*BrARF4.2* (BraA02g010280.3C)	chr02: 5008076..5008416
Small RNA pathway	*AGO1*	AT1G48410	*BrAGO1.1* (BraA05g020200.3C)	chr05: 13144642..13150296
	*AGO7*	AT1G69440	*BrAGO7* (BraA07g030370.3C)	chr07: 22224668..22227518
	*AGO10*	AT5G43810	*BrAGO10.1* (BraA06g043560.3C)	chr06: 28421795..28417452
			*BrAGO10.2* (BraA09g019960.3C)	chr09: 12912026..12912769
	*SGS3*	AT5G23570	*BrSGS* (BraA06g031000.3C)	chr06: 21377862..21380043
	*RDR6*	AT3G49500	*BrRDR6* (BraA01g023640.3C)	chr07: 13537572..13541477
	*HYL1*	AT1G09700	*BrHYL1.1* (BraA06g006590.3C)	chr06: 3729485..3727977
			*BrHYL1.2* (BraA08g032470.3C)	chr08: 21597984..21599798
	*DCL1*	AT1G01040	*BrDCL1* (BraA10g000840.3C)	chr10: 428538.423842
	*DCL4*	AT5G20320	*BrDCL4* (BraA10g020250.3C)	chr10: 14712105.14721149
	*SE*	AT2G27100	*BrSE1* (BraA07g018000.3C)	chr07: 15424372.15427821
			*BrSE2* (BraA04g019460.3C)	chr04: 14704649.14701441

### Motif Display and Phylogenetic Analyses of Genes Responsible for Leaf ad-ab Polarity in Chinese Cabbage

To search the conserved motifs of the proteins, the amino acid sequences of the 41 genes responsible for leaf ad-ab polarity in Chinese cabbage were uploaded and analyzed using the online tool MEME Suite 5.1.1^2^ (Bailey et al., 2009). The amino acid sequences of these genes in *A. thaliana* were downloaded from the TAIR database^3^ (Rhee et al., 2003). The ClustalW 2.0 (Larkin et al., 2007) and MEGA6.0 (Tamura et al., 2013) software were used to construct the phylogenetic tree. The analysis of the conserved motifs was conducted using Pfam^4^ (Sara et al., 2019) and SMART (Letunic et al., 2009). The analyses of collinearity and visualization for the genes responsible for leaf ad-ab polarity were done using MCScan X (Wang et al., 2012) and TBtoolse^5^ (Chen et al., 2020), respectively.

### SSR Identification and Primer Design

Based on the target gene and its upstream (5 kb) and downstream (5 kb) sequences (Gong et al., 2014). SSR primers were identified and located by MIcroSAtellite (MISA) (Thiel et al., 2003) software with Perl. The search criteria was set to be as follow: ≥ten repeat units for mononucleotides, ≥seven repeat units for dinucleotides, and ≥five repeat units for tri-, tetra-, penta-, and hexanucleotides. The interrupted compound SSRs were also listed as search targets when the interval was less than or equal to 10 bp. Primer pairs were designed based on the sequences of the identified SSR using Primer 3.0 with Perl. The parameters of Primer 3.0 were set as follows: (a) melting temperature between 55°C and 65°C; (b) GC content between 40% and 60%; (c) primer length between 18 and 27 bases; and (d) PCR products length between 100 and 300 bp. The other parameters were set with default values. 969 SSR primer pairs were designed in all.

### DNA Extraction

DNA was extracted from young leaves of the F_2_ population by the CTAB method (Murray and Thompson, 1980; Rogers and Bendich, 1985). 5–10 μL genomic DNA was used to assess the sample quality using 1.0% agarose gel. A NanoDrop2000 spectrometer was used to assess the DNA quality and concentration.

### Assessment of SSR Polymorphisms

The validation of polymorphic primers was done using 12 Chinese cabbage accessions with different heading traits selected from the F_2_ population. 362 pairs of SSR primers were selected from the newly designed primers and were used to detect the SSR polymorphisms among the 12 accessions. The primers were synthesized by Sangon Biotech, Shanghai, China. A total volume of 10.0 μL was used to perform PCR, including 1.0 μL of genomic DNA (50 ng/μL), 0.8 μl of dNTPs (2.5 mmol/L), 0.5 μL of each of forward and reverse primers (50 ng/μL), 1 μL of 10 × PCR buffer (Mg_2_^+^), 0.1 μL of Taq DNA polymerase (2.5 U/μL, TaKaRa, Dalian, China), and 6.1 μL H_2_O. The reactions conditions were as follows: 3 min at 94°C; then 35 cycles of 45 s at 94°C, 30 s at the proper annealing temperature, 45 s at 72°C, and a final elongation 5 min at 72°C. After the PCR, the products were separated using 7% denaturing polyacrylamide gels and visualized by silver nitrate staining.

### Genetic Map Construction

The markers to construct the F_2_ genetic map were selected from 581 insertion-deletion (InDel) markers, 60 single nucleotide polymorphism (SNP) markers, 123 random SSR markers, and 362 ad-ab polarity related SSR markers. The genotypes of parents “CC-48” and “PC-101” were screened using the 60K *B. napus* array developed by array TraitGenetics (Germany), resulting in 5,795 polymorphic SNPs. The SNP markers were selected from the TraitGenetics dataset. The BRAD database were used to design the InDel markers and Primer Premier 5.0 (Lalitha, 2004) were used to design the random SSR markers. Native-PAGE method were used to analyze the genotypes of F_2_ population with InDel and SSR markers. A 96-well LightScanner instrument was used for SNP genotyping by high-resolution melting analysis of small amplicons.

The genotypes data were classified as type “a” or “b” based on whether they were the parents of “14Q-141” or “14Q-279,” the undefined and missing data were showed by “−.” JoinMap version 4.0 software was used to construct genetic maps for F_2_ populations (Van Ooijen, 2006). After creating the population nodes, the markers were assigned into the linkage groups (LGs) based on the LOD value of 8.0–10.0. With the method of Kosambi, frequencies of recombination were transformed into centiMorgans (cM) to calculate genetic distance. Mapchart 2.32 was used to draw the map (Voorrips, 2002). A comparison map of genetic linkage distance and physical position was constructed using the ALLMAPS software^6^ (Tang et al., 2015).

## Results

### Classification and Collinearity Analysis of Genes

**FIGURE 2:**
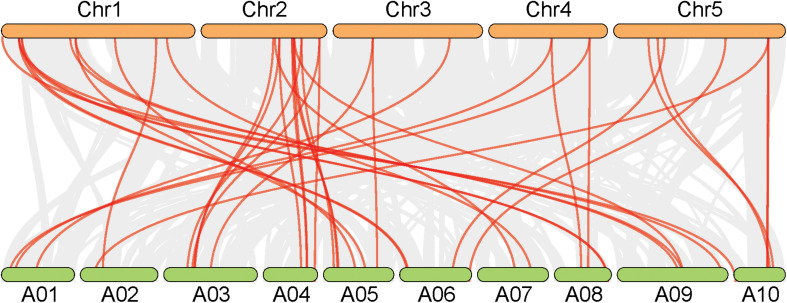
Synteny analysis of leaf ad-ab polarity–controlling genes between Chinese cabbage and *Arabidopsis*.

**FIGURE 3:**
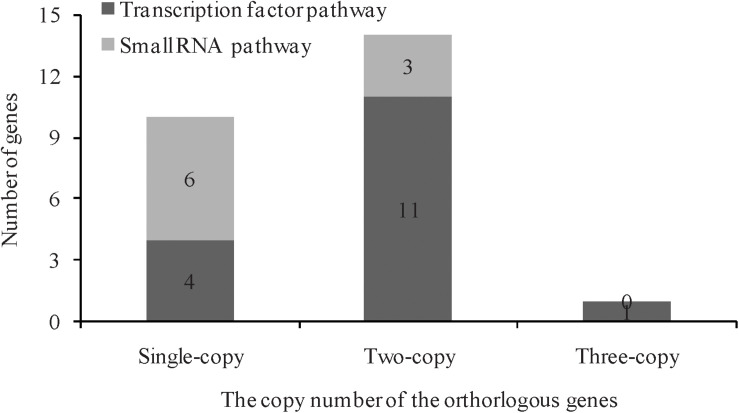
The copy number of orthorlogous of leaf ad-ab polarity–controlling genes between *Arabidopsis* and Chinese cabbage.

In *A. thaliana*, a total of 26 genes responsible for leaf ad-ab polarity were identified, including genes of transcription factors and small RNA pathways (Liang et al., 2016). By comparing with these *Arabidopsis* genes, 41 orthologs genes were detected in Chinese cabbage, including 10 transcription factors for adaxial determination, 10 transcription factors for middle domain determination, 9 transcription factors for abaxial determination, and 12 small RNAs for ad-ab polarity. According to the genomic sequence information of *A. thaliana* and Chinese cabbage, orthologous genes related to leaf ad-ab polarity establishment, except for AT4G00180, were all obtained in Chinese cabbage. These genes includes 10 single copies, 14 double copies, and one triple copies (Figure 2). A total of four single copies, 11 double copies, and one triple copies were detected in the transcription factor pathway, with the proportion of single copy to multiple copies being 1:3. In the small RNA pathway, a total of six single copies, three double copies, and no triple copies were detected, with the proportion of single copy to multiple copies being 2:1 (Figure 3). 60% of the genes exist as double or triple copies in Chinese cabbage.

### Distribution of the Genes Responsible for the Leaf ad-ab Polarity and the Characterization of SSR Loci in Chinese Cabbage

**FIGURE 4:**
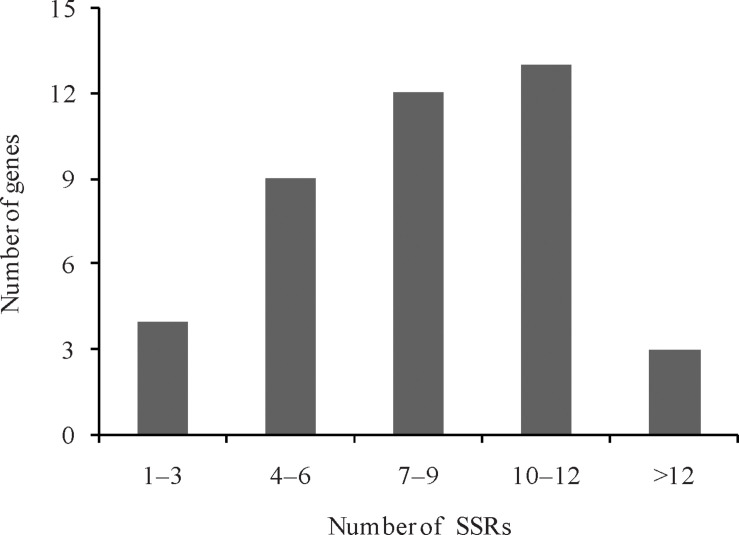
Number of SSRs in leaf ad-ab polarity–controlling genes in Chinese cabbage.

Genes responsible for the leaf ad-ab polarity are distributed on 10 chromosomes in Chinese cabbage, with most of them (6, 14.63%) occurring on chromosome A05 and only a very few (2, 4.88%) on chromosome A01. Genes on other chromosomes are distributed evenly. A total of 341 SSR loci were developed for the genes responsible for leaf ad-ab polarity in Chinese cabbage. Among the 41 genes, four contain 1–3 SSR loci, nine contain 4–6 SSR loci, 12 contain 7–9 SSR loci, 13 contain 10–12 SSR loci, and only three contain more than 12 SSR loci (Figure 4). 55 SSR loci are located within the genes. According to the principle of SSR primer design, 323 SSR loci were used to design the corresponding SSR primers. A total of 362 SSR primer pairs were selected for this study.

### Structure and Motif Composition of Genes Responsible for Leaf ad-ab Polarity in Chinese Cabbage

**FIGURE 5:**
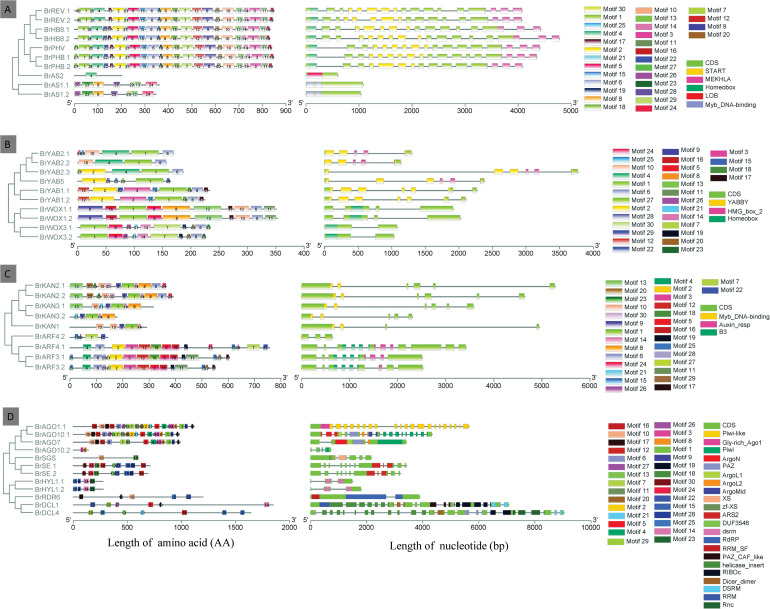
The gene structure and motif analyses of leaf ad-ab polarity–controlling genes in Chinese cabbage. The exon-intron structure of leaf ad-ab polarity–controlling genes were showed on the left, and schematic diagram of amino acid motifs of leaf ad-ab polarity–controlling proteins were showed on the right. **(A)** Adaxial-controlling genes in Chinese cabbage; **(B)** Middle domain-controlling genes in Chinese cabbage; **(C)** Abaxial-determinant genes in Chinese cabbage; **(D)** Small RNA-controlling genes in Chinese cabbage.

The exon-intron organization of all the identified genes responsible for leaf ad-ab polarity was analyzed to get more insight into their evolution in Chinese cabbage. As shown in Figure 5, the length of the genes responsible for leaf ad-ab polarity is mostly within 6 kb, with only *BrDCL1* and *BrDC*L4 reaching 7 and 9 kb, respectively. All the genes possess 1–25 exons. Genes *BrAS2*, *BrAS1.1*, and *BrAS1.2* have only one exon and genes *BrDCL4* has 25 exons. The distributions of exons and sequence length among these genes present obvious distinctions. A schematic representing the structure of four different regulatory pathway proteins is shown in Figure 5; the 30 motifs are named Motifs 1–30. Motifs 1, 6, and 10 are widely distributed domains. The members in the same groups are usually composed by a similar motif composition. For example, *BrREV*, *BrPHB*, *BrPHV*, and *BrATHB8* in the adaxial determinant pathway contain relatively more motifs and show a highly similar motif distribution. The number of motifs varies greatly. In adaxial determinant pathway, genes *BrPHV*, *BrPHB.1*, and *BrPHB.2* have the largest number of motifs (24), whereas gene *BrAS2* contains the fewest motifs, only Motif 4. Among the homologous genes *BrARF4.1*, *BrARF4.2*, *BrAGO10.1*, and *BrAGO10.2*, the conserved motifs of one gene are partially lost in another. A total of two loci are located in the START conserved domain, one in gene *BrPHV* and the other in *BrREV1*.

### SSR Loci Analysis of the Genes Responsible for Leaf ad-ab Polarity in Chinese Cabbage

The repeat-type distributions of 341 SSR loci are shown in Table 2. Repeat types of SSR loci of the genes responsible for leaf ad-ab polarity are abundant in Chinese cabbage. Five repeat types are identified: mononucleotide (231) are the most abundant repeats (67.74%), and the following are dinucleotides (19.65%) and trinucleotides (9.38%); pentanucleotides and hexanucleotides are the fewest (0.30%, respectively). No tetranucleotides are found in the repeats. A/T, AT/TA, AAG, CAATG, and TAGATA are the predominant motifs. Mononucleotide repeats contain seven double-mononucleotide motifs, (A)_11_g(A)_10_, (G)_10_tgtagc(T)_16_, (A)_10_tttatc(T)_11_, (T)_15_(G)_12_, (T)_10_attttgg(A)_10_, (A)_10_gttcac(T)_11_, and (A)_10_tt(A)_13._ Dinucleotide repeats contain two double-dinucleotide motifs, (TG)_10_(TA)_8_ and (TC)_9_(TA)_8_.

**TABLE 2:** Type, number and frequency of SSRs from leaf ad-ab polarity–controlling genes in Chinese cabbage.

Repeat types	Types of repeat motif	Total number of repeat motif	Percentage(%)	Type of predominant motif
Mononucleotide	4	238	69.79	T/A
Dinucleotide	8	69	20.23	TA/AT
Trinucleotide	26	32	9.38	AAG
Tetranucleotide	0	0	0	–
Pentanucleotide	1	1	0.30	GAATG
Hexanucleotide	1	1	0.30	TAGATA
Total	40	341	100	

### Screening and Polymorphic Analysis of SSR Primers

362 pairs of primers were selected at random from the 969 pairs for evaluation using 12 Chinese cabbage accessions with different heading types. The results show that clear amplification products can be obtained by 213 (58.84%) primer pairs, of which 89 (24.59%) primer pairs resulted in polymorphic bands (Supplementary Figure S1A), and 68 (18.78%) primer pairs gave no polymorphism (Supplementary Figure S1B). In addition, 149 (41.16%) primer pairs failed to give any amplification products (Supplementary Figure S1C), and 56 (15.47%) primer pairs resulted in non-specific bands (Supplementary Figure S1D).

Among the 89 polymorphic SSR primers, 23 pairs with high polymorphism and clear bands were selected to analyze the 214 Chinese cabbage accessions. These SSR markers distribute on chromosomes A01, A02, A03, A04, A05, A06, A08, A09, and A10. These primers amplified 53 polymorphic bands, and the numbers of amplified fragments varied from two to four. On average, each primer amplifies 2.3 bands. Two alleles were detected by 17 SSR primers, three alleles by five SSR primers, and four alleles by one SSR primer. The primer S119 located on chromosome A05 detect the highest number of alleles (four). 78.6% of the accessions were amplified with one allele (Supplementary Table S1).

### Genetic Map Construction

**FIGURE 6:**
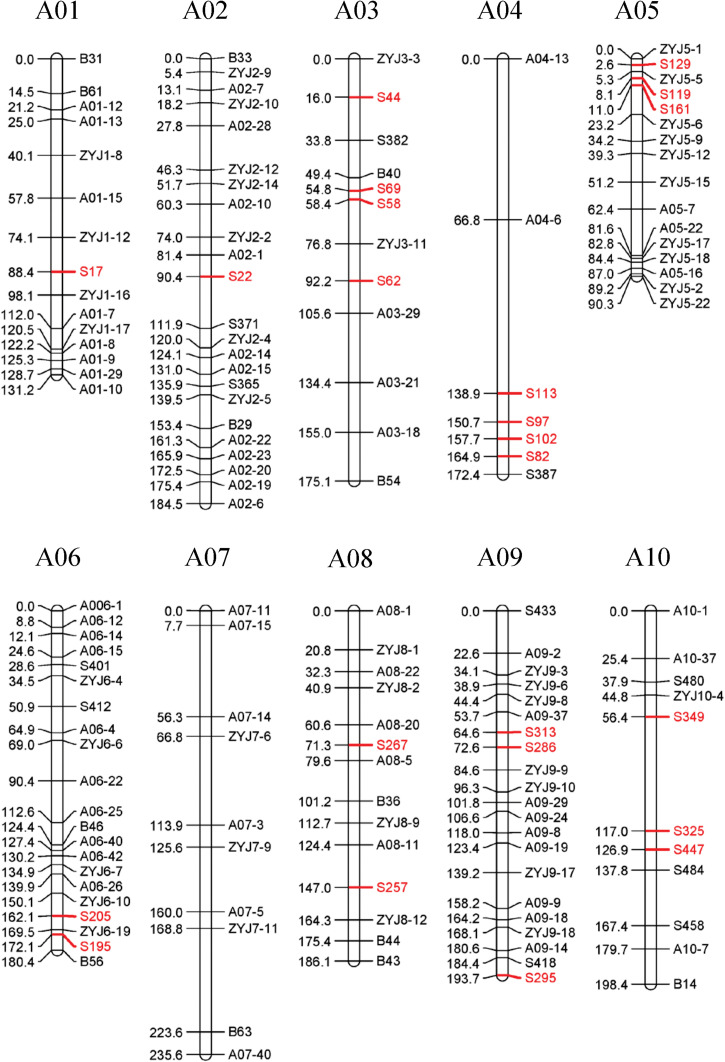
A genetic linkage map of the F_2_ population. Recombination distances (cM) are showed on the left, marker names on the right of each linkage group.

**FIGURE 7:**
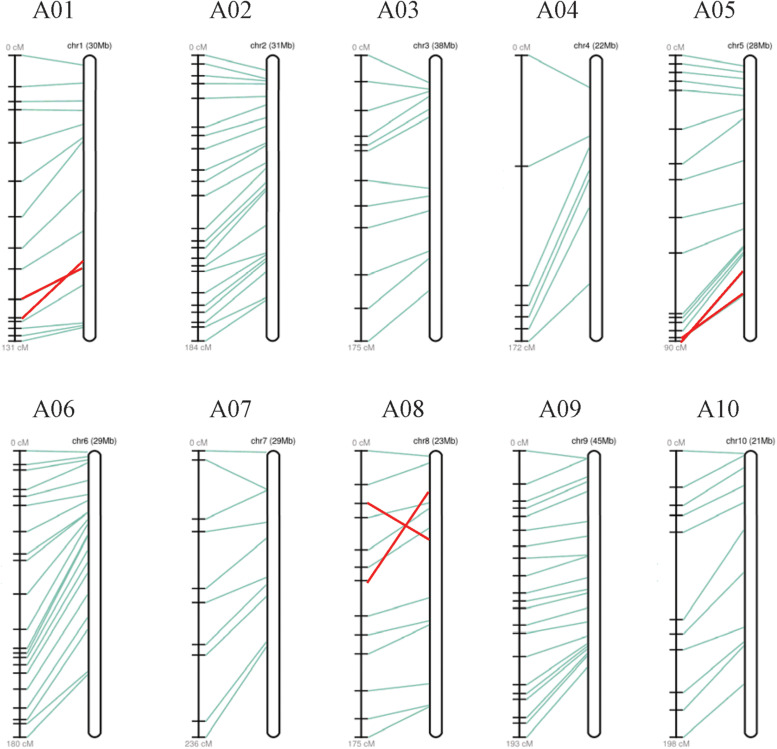
A genetic linkage map and a physical map constructed based on F_2_ population. In each group, recombination distances (cM) are showed on the left, physical distances (Mb) are showed on the right, and their correspondences are showed with lines in the middle.

34 SSRs (23 specific SSR primers and 11 random SSR primers), 13 SNPs, and 103 InDel markers distributed over 10 linkage groups (LGs) were used to construct the linkage map. The total map length is 1747.57 cM with an average distance of 11.65 cM between adjacent markers (Figure 6). The most markers (23) were in linkage group (LG) A02 and the fewest (7) in LG A04. The length of each LG ranged from 90.32 to 235.61 cM. The biggest gap in the genetic map was 24.63 cM in LG A04, and the smallest gap was 5.65 cM in LG A05. The result showed that the sequences of markers in genetic linkage map and physical map are generally consistent, with only three groups of markers distribute in the LG A01, A05, and A08 showing different sequences (Figure 7).

## Discussion

### Analysis of the Evolution of the Relationship Between SSR Loci, Gene Structure, and Conserved Motifs

During plant growth and development, the leaf shapes of different species and the same plant at different growth stages may show diverse morphology. It was found that the leaf ad-ab patterning can affect leaf curvature (Kidner and Timmermans, 2010; Yamaguchi et al., 2012). Based on 26 genes responsible for leaf ad-ab polarity in *A. thaliana*, we identified 41 orthologous genes in Chinese cabbage by a comparative genomic analysis. 75.6% of these genes contains double or triple copies in Chinese cabbage, indicating that they went through duplication and were reserved after whole genome triploidization. Multiple copy genes were also detected in the research of genes for glucosinolate (GS) biosynthesis in Chinese cabbage (Gong et al., 2014). However, among 12 orthologs genes detected in the small RNA pathway responsible for leaf ad-ab polarity, 42% genes have one or no copies in Chinese cabbage, and none has more than two copies. It was speculated that Chinese cabbage may have experienced a triplication of the whole genome and then undergone diploidy to delete one or two gene copies (Cheng et al., 2012; Tang et al., 2012). We inferred that there was a large amount of gene lost or rearranged during this process (Wang et al., 2011; Cheng et al., 2013). It remains to be further verified whether the leaf morphology establishment is affected by the different copies of leaf ad-ab polarity genes in various breeding materials.

Gene structure analysis is useful in mining the relationship between gene family evolution and gene duplication. The number and distribution of introns and exons may be related to gene evolution. We found that obvious differences exist in the distribution of exons and sequence length among four group of genes. We speculate that these genes may play distinct roles in leaf development. Through motif analysis, we found that Motifs 1, 6, and 10 are widely distributed domains, suggesting that these domains may be very important for the gene function. Between the homologous genes *BrARF4.1*, *BrARF4.2*, *BrAGO10.1*, and *BrAGO10.2*, the conserved motifs of one gene was partially lost in another, suggesting a possibility of fragments lost during evolution. The striking dissimilarity between four groups of genes provides useful evidence for the study of genome duplication and phylogenetic evolution.

### Development of Specific SSR Markers for Genes Controlling Leaf ad-ab Polarity

Simple sequence repeats markers are widely used because they are simple, rapid, low-cost, and have good repeatability. In molecular-assisted selection breeding, analyzing the diversity by using the SSR markers based on the target functional gene sequences or their upstream and downstream regions can overcome the problem of identification bias for non-gene region. In this study, 341 SSR loci were developed based on the 41 genes responsible for leaf ad-ab polarity, with the mononucleotide repeats (242, 70.96%) the most common and showing a strong bias toward A/T. 969 specific primers were designed from these SSR loci. These studies laid a foundation for further research on head-related traits in Chinese cabbage.

### Application of Specific SSR Markers

Simple sequence repeats markers with wide genomic distribution and a high degree of polymorphism are widely used in genetic analyses. A total of 6 jujube cultivars and wild types were used to verify the polymorphism of 1000 SSR primers; it was observed that, among the germplasms, 725 pairs (72.5%) were clear and effective and 511 pairs (51.1%) were polymorphic (Xiao et al., 2015). Eight Chinese cabbage inbred lines were used to screen the polymorphism of 77 SSR primers; it was shown that 49 SSR primers (63.64%) could amplify clear bands and 16 of them (20.8%) were polymorphic (Gong et al., 2014). In this study, 12 Chinese cabbage accessions with different heading types were used to evaluate the polymorphism of 362 SSR primer pairs, and 23 pairs (6.35%) resulting in high polymorphism and clear bands were selected to construct the linkage map. QTL analysis for 4 leafy head traits of Chinese cabbage was performed, which were head top shape (HTS), Head height (HH), Head Weight (HWe), Plant weight (PWe). The result showed that the sequences of markers in the linkage map were generally consistent with that of the physical map, 12 QTLs were mapped including 6 QTLs with 7 specific SSR coseparation, indicating that these specific SSR markers can be used in the further study and the candidate genes could be quickly indentified with them. However, the polymorphism of these SSRs was low, possibly because the genes responsible for leaf ad-ab polarity were highly conserved during evolution. Therefore, development of primers from other SSR loci of functional genes and other molecular markers such as InDel and SNP are still needed.

As the gene sequences are conserved between Chinese cabbage and other *Brassica* plants, SSR primers from Chinese cabbage are transferable to relative species. In our study, 55 SSR loci were located with the genes and two were in START conserved domain. The molecular genetic map constructed by these genes specific SSR markers, especially markers within the genes, can provide reference for linkage and QTL mapping of target genes among related species. Furthermore, as the reference markers, these SSR markers can be used to integrate the molecular genetic maps among relative species, thus providing a new method for comparative genomics research.

## Conclusion

Establishment of leaf ad-ab polarity is tightly related to leaf incurvature in Chinese cabbage, which is a main factor result in the formation of a leafy head. In this study, by comparing the sequences of genes controlling leaf ad-ab polarity in *A. thaliana*, 41 candidate orthologous genes were found in Chinese cabbage. Based on these gene, 341 SSRs were detected and characterized. A genetic linkage map were constructed by SSR and other molecular markers screened. The result showed that the sequences of markers in genetic linkage and physical map was generally consistent, suggesting that these SSR markers were valuable for assisted-selective breeding in Chinese cabbage.

## Data Availability Statement

All datasets presented in this study are included in the article/Supplementary Material.

## Author Contributions

YG and YL performed the research and wrote the manuscript. XL, NL, and XS surveyed the morphological characteristics. XZ and DF performed the assessment of SSR polymorphisms. ML, SX, and AG performed the motif display and phylogenetic analyses of genes. YW and XC performed the genetic map construction analysis. NL and JZ reviewed the manuscript. JZ and SS designed the research and reviewed the manuscript. All authors contributed to the article and approved the submitted version.

## Conflict of Interest

The authors declare that the research was conducted in the absence of any commercial or financial relationships that could be construed as a potential conflict of interest.

## Footnotes

1 http://brassicadb.org/brad/

2 http://meme-suite.org/

3 http://www.arabidopsis.org/

4 http://pfam.xfam.org/

5 https://github.com/CJ-Chen/TBtools

6 https://github.com/tanghaibao/jcvi/wiki/ALLMAPS
