# Supplementary figures and images for: Development and Application of SSR Markers Related to Genes Involved in Leaf Adaxial-Abaxial Polarity Establishment in Chinese Cabbage (Brassica rapa L. ssp. pekinensis)
Source: Front Genet. 2020 Jul 23;11:773. doi: 10.3389/fgene.2020.00773 (PMC7391075; doi:10.3389/fgene.2020.00773)

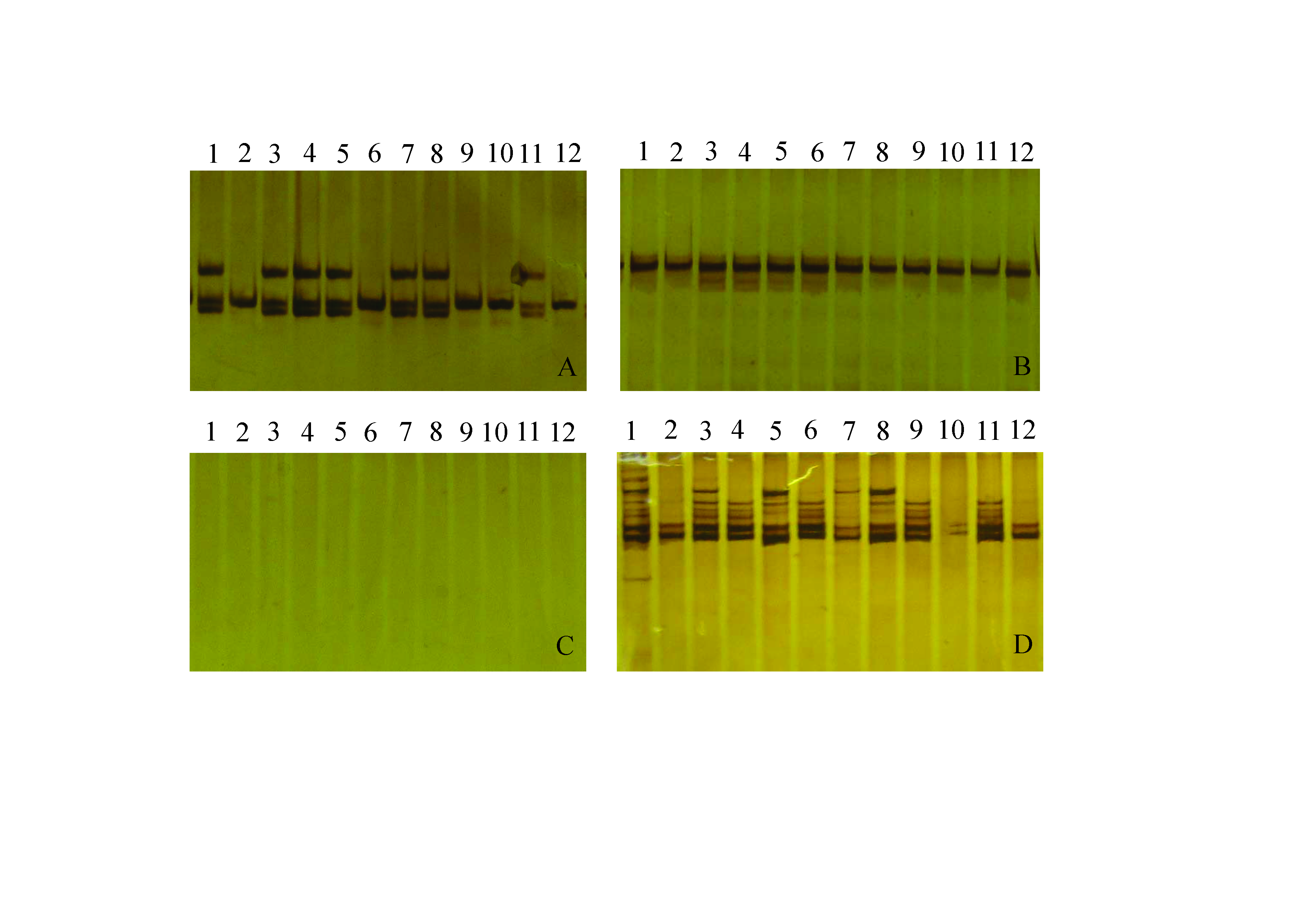

Supplement: Supplementary file 1 [file Image_1.TIF]
